# Evaluation of Ammonia Nitrogen Exposure in Immune Defenses Present on Spleen and Head-Kidney of Wuchang Bream (*Megalobrama amblycephala*)

**DOI:** 10.3390/ijms23063129

**Published:** 2022-03-15

**Authors:** Honghui Guo, Siqi Chen, Kang Ouyang, Yu Kuang, Hui Yang, Yingying Wang, Rong Tang, Xi Zhang, Dapeng Li, Li Li

**Affiliations:** 1 College of Fisheries, Huazhong Agricultural University, Wuhan 430070, China; honghuiguo@webmail.hzau.edu.cn (H.G.); ouyangkang@webmail.hzau.edu.cn (K.O.); kuangyu@webmail.hzau.edu.cn (Y.K.); yanghui1325196@yeah.net (H.Y.); wangyingying0219@163.com (Y.W.); tangrong@mail.hzau.edu.cn (R.T.); zhangxi@mail.hzau.edu.cn (X.Z.); ldp@mail.hzau.edu.cn (D.L.); 2 Hubei Aquaculture Technology Extension Center (Hubei Aquatic Breeds Introduction and Breeding Center), Department of Agriculture and Rural Affairs of Hubei Province, Wuhan 430060, China; m185762552@163.com; 3 Engineering Research Center of Green Development for Conventional Aquatic Biological Industry in the Yangtze River Economic Belt, Ministry of Education, Wuhan 430070, China; 4 Hubei Provincial Engineering Laboratory for Pond Aquaculture, Wuhan 430070, China; 5 Freshwater Aquaculture Collaborative Innovation Center of Hubei Province, Wuhan 430070, China

**Keywords:** ammonia, stress, toll-like receptors, spleen, head-kidney, immunity

## Abstract

Ammonia is one of the most important environmental factors in aquatic ecosystems. However, there are limited studies on the effects of chronic or long-term ammonia stress and its potential molecular mechanism in fish. This study aimed to investigate the immune response and molecular mechanisms in the spleen and head-kidney of fish following chronic ammonia exposure. *Megalobrama amblycephala* (9.98 ± 0.48 g) were exposed to different concentrations of total ammonia nitrogen (0–30 mg/L) for 30 days. Ammonia exposure caused significant increases in cortisol levels and decreases in lysozyme and complement 3/4 concentrations in the serum, indicating inhibitory effects of ammonia stress on innate immune responses. Ammonia exposure also induced concentration-dependent increases in ammonia concentrations in tissue, pathological damage and indexes of spleen and head-kidney. Additionally, the contents of immunoglobulin M (IgM), interleukin 1β (IL-1β) and tumor necrosis factor α (TNF-α) as well as mRNA levels of toll-like receptors (TLRs)/Myeloid differentiation factor 88 (MyD88)-independent signaling molecules in the spleen and head-kidney were significantly downregulated after ammonia exposure. Our findings suggested that chronic ammonia exposure caused the suppression of innate and adaptive immune responses through downregulating TLR/MyD88-independent signaling. Adverse influences of chronic ammonia stress were more severe in the spleen than in the head-kidney.

## 1. Introduction

Ammonia is one of the most important environmental factors in aquatic ecosystems that affects the growth and health of aquatic animals [1]. Ammonia nitrogen generally comes in one of two ionized forms—NH_4_^+^ and the un-ionized form, NH_3,_ in the aquatic environment. NH_3_ is extremely toxic to fish by reason of its ability to diffuse across cell membranes easily [2,3,4]. Over the past decades, high levels of ammonia frequently occur in aquatic environments due to disorderly discharges of sewage effluent, agricultural run-off, and high-density aquaculture [5,6,7]. Although the recommended level of ammonia nitrogen in drinking water is up to 3 mg/L (NH_4_/L) [8], the real ammonia level can reach much higher (>20 mg/L) at times, due to ineffective or nonexistent sewage treatment or other reasons [9,10].

In fact, the immunosuppressive effects of ammonia in fish have been proven to contribute to disease outbreaks [11,12]. In some studies on *Takifugu rubripes*, *Scophthalmus maximus* and *Pelteobagrus fulvidraco*, acute ammonia exposure could upregulate gene expression levels of B-cell activating factor, heat shock protein 70/90, *tnf-α*, and interleukin-1/1β/6/8/12 [13,14,15]. Wang et al. also reported that serum lysozyme activities were decreased significantly in *Acanthopagrus schlegelii* after 24 h of ammonia exposure [16]. Similarly, decreased mRNA levels of complement C3 and IgM were observed in the spleens of *Pelteobagrus vachellii* and *Rhynchocypris lagowski* under acute ammonia exposure [17,18]. Although much attention has been paid to the effects of acute ammonia exposure on fish immunity, there are only limited studies on the effects of chronic or long-term ammonia stress and its potential molecular mechanism. In addition, some investigators have reported adverse effects of ammonia nitrogen on mRNA expression of TLRs through transcriptional analysis [16,19,20,21,22]. Consequently, we hypothesize that persistent ammonia exposure also could affect fish immunity, in which the TLR signaling pathway might play an important role.

The spleen and head-kidney are the major immune organs in teleost fishes, which are responsible for trapping and clearing foreign particulate materials and maintaining a stable internal environment [23,24]. They are also the main sites where immune antibodies are produced [25,26,27]. Additionally, in fish, the innate immune system consists of fixed and mobile cells as well as a wide range of defense molecules (such as lysozyme, complement, cytokines) [28,29]. Wuchang bream (*Megalobrama amblycephala*) is a cyprinid fish native to the Yangtze basin of China, which was ranked 11th in the world in total annual production of this fish in 2014 [30]. As the main aquaculture species, *M. amblycephala* is a relatively sensitive species to ammonia nitrogen and often suffers from stress induced by ammonia nitrogen [31,32,33]. In light of the above, a chronic exposure experiment was conducted in Wuchang bream to elucidate the features of immune responses after chronic ammonia exposure and the mechanism behind these effects through the detection of changes in serum immune parameters, histology, and TLR pathway-related molecules in the spleen and head-kidney. Our results are also conducive to monitoring the health status and welfare of *M. amblycephala* in intensive aquaculture systems.

## 2. Results

### 2.1. Ammonia Content in Spleen and Head-Kidney

**Figure 1:**
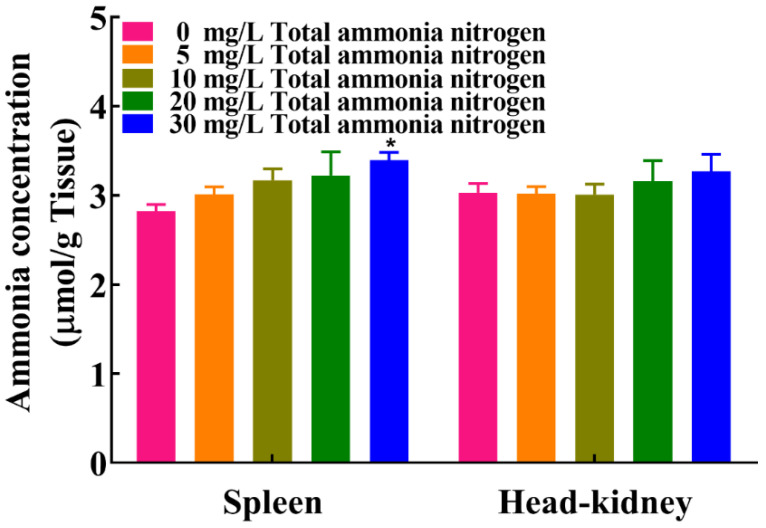
Effects of chronic ammonia exposure on ammonia accumulation in the spleen and head-kidney of *M. amblycephala*. Each column represents mean ± SE of six duplicates. The values of *p* < 0.05 are represented as “*” above the column, indicating significant difference versus control, respectively.

Figure 1 showed ammonia content in the spleen and head-kidney of *M. amblycephala* after 30 days of exposure to various levels of ammonia. A significant increase in ammonia level was observed in the spleen of fish exposed to 30 mg/L total ammonia nitrogen compared with the control group (*p* < 0.05). There was no statistical difference in ammonia levels in head kidney between all ammonia treatment groups and control group (*p* > 0.05).

### 2.2. Serum Cortisol, Lysozyme, C3 and C4 Levels

**Figure 2:**
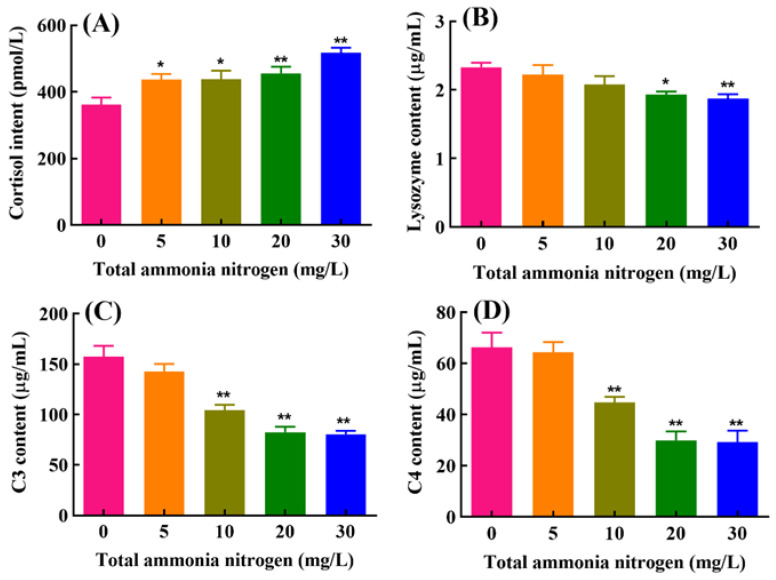
Effects of chronic ammonia exposure on levels of serum cortisol, lysozyme, C3 and C4 (**A**–**D**) in *M. amblycephala*. Each column represents mean ± SE of six duplicates. The values of *p* < 0.05 and 0.01 are represented as “*” and “**” above the column, indicating significant difference versus control, respectively.

As showed in Figure 2, serum cortisol levels increased significantly in all ammonia treatment groups compared with the control group (*p* < 0.05). Contrarily, serum lysozyme activities were significantly decreased in the fish exposed to higher concentrations of ammonia (20 and 30 mg/L total ammonia nitrogen) (*p* < 0.05). Complement C3 and C4 levels in the serum were significantly reduced in the groups treated with 10 to 30 mg/L total ammonia nitrogen (*p* < 0.01).

### 2.3. Immunity Organ Indexes

**Figure 3:**
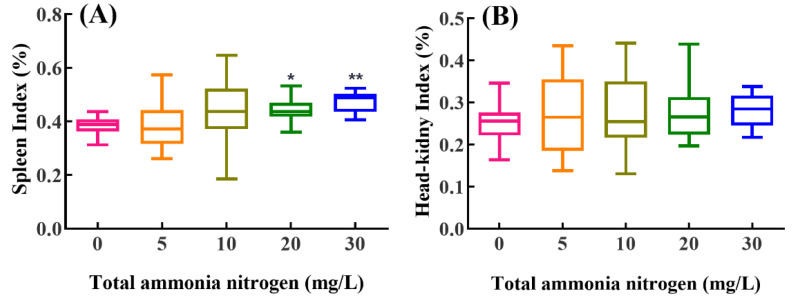
Effects of chronic ammonia exposure on spleen (**A**) and head-kidney indexes (**B**) in *M. amblycephala*. Values are expressed as Min to Max (*n* = 18). The values of *p* < 0.05 and 0.01 are represented as “*” and “**” above the column, indicating significant difference versus control, respectively.

Both the spleen and head-kidney indexes increased with the increase in total ammonia nitrogen concentrations after exposure (Figure 3). Compared with the control group, the spleen index was significantly elevated in the fish treated with higher total ammonia nitrogen (20 and 30 mg/L) (*p* < 0.05). No significant difference was detected in the head-kidney index between all ammonia treatment groups and control group (*p* > 0.05).

### 2.4. Pathological Evaluation

**Figure 4:**
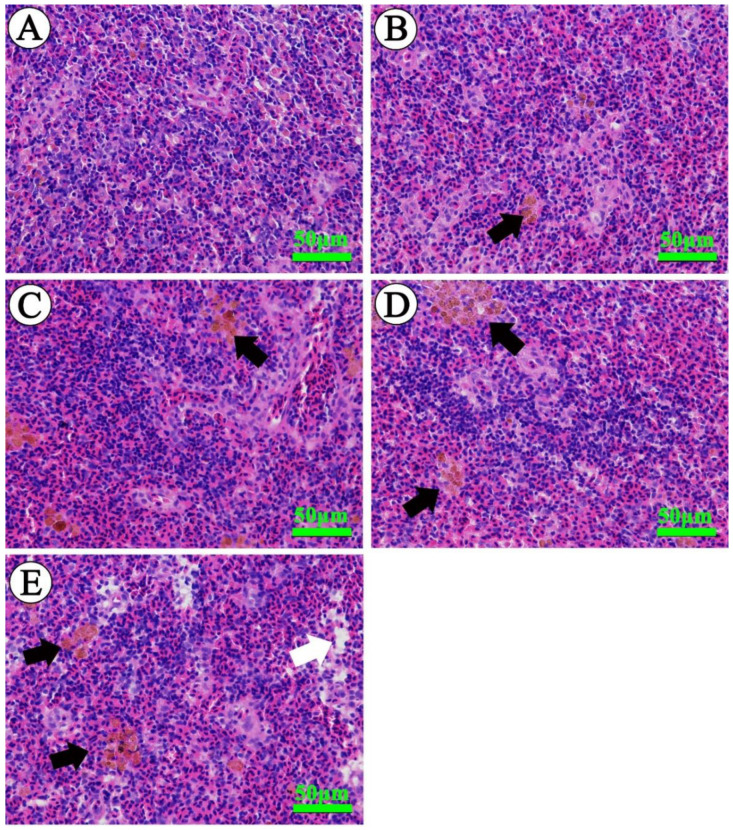
Light microscope photographs of spleens of *M. amblycephala* (H&E). (**A**) Spleen tissue of control fish showing normal architecture. (**B**–**D**) Spleen tissues from fish exposed to 5, 10 and 20 mg/L ammonia nitrogen showing an increase in erythrocytes and melano-macrophage centers (black arrows). (**E**) Spleen tissue from fish exposed to 30 mg/L ammonia nitrogen showing increased melano-macrophage centers (black arrows) as well as cytoplasm vacuolization (white arrows). Scale bar = 50 μm.

The spleen of Wuchang bream in the control group showed a normal appearance with abundant erythrocytes and leukocytes (Figure 4A). After exposure to ammonia for 30 days, slight increases in the numbers of erythrocytes were noted along with the occurrence of melano-macrophage centers in the spleen of fish exposed to 5 mg/L total ammonia nitrogen (Figure 4B, Table 1). Similar but more serious changes were observed in fish treated with 10 to 30 mg/L total ammonia nitrogen, such as increases in the number and size of melano-macrophage centers as well as markedly increased erythrocyte numbers (Figure 4C,D). In addition, cytoplasm vacuolization was also detected in the spleen of fish exposed to 30 mg/L total ammonia nitrogen (Figure 4E, Table 1).

**Figure 5:**
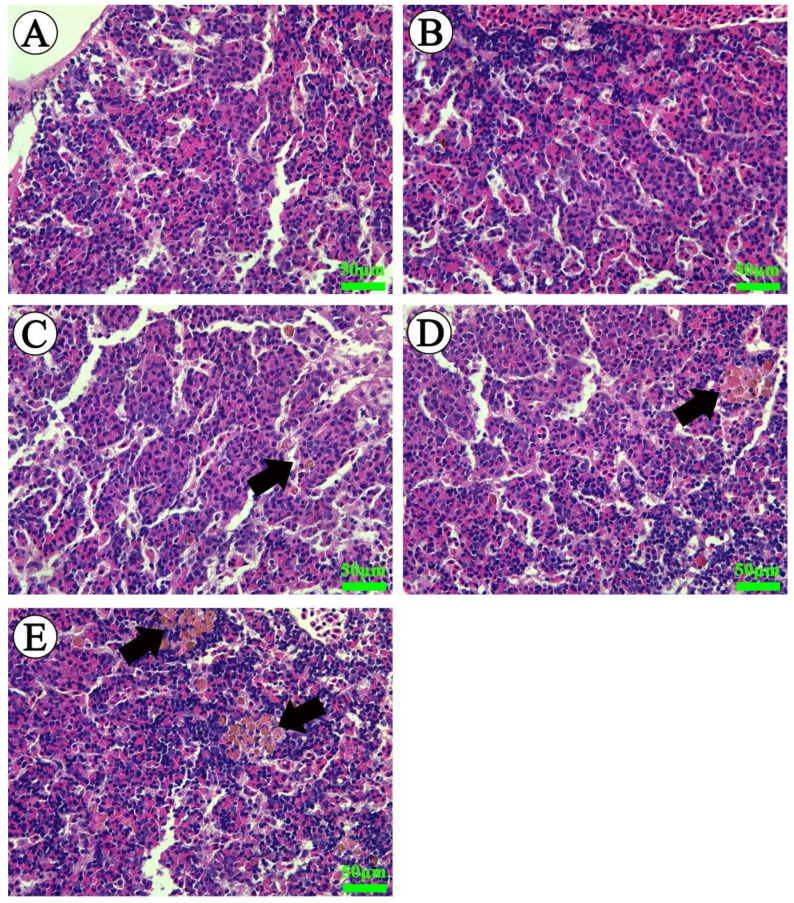
Light microscope photographs of head-kidney tissue of *M. amblycephala* (H & E). (**A**,**B**) The head-kidney of fish exposed to 0 and 5 mg/L ammonia nitrogen showing normal structure. (**C**–**E**) The head-kidney from fish exposed to 10, 20 and 30 mg/L ammonia nitrogen showing the increase in melano-macrophage centers in number and size (black arrows). Scale bar = 50 μm.

As for the head-kidney, Figure 5A shows a normal structure of head-kidney tissue in control fish after exposure. No obvious pathological sign was noted in the head-kidney of fish exposed to lower total ammonia nitrogen (5 mg/L) (Figure 5B, Table 1). Slight increases in the number of melano-macrophage centers were observed in fish exposed to 10 and 20 mg/L total ammonia nitrogen (Figure 5C,D, Table 1). The size and number of melano-macrophage centers were enhanced in the 30 mg/L total ammonia nitrogen group (Figure 5E, Table 1).

### 2.5. Tissue Immune Parameter Analysis

**Figure 6:**
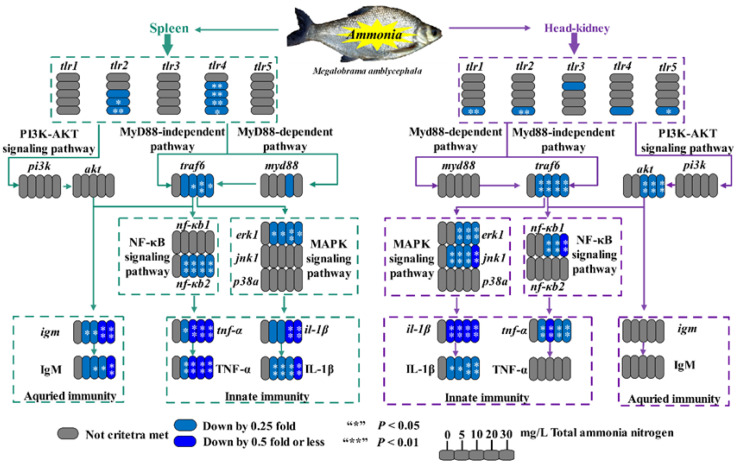
Relative mRNA levels of TLR signaling pathway genes as well as the contents of TNF-α, IL-1β and IgM in the spleen and head-kidney of *M. amblycephala*. Values expressed as means ± SE of six duplicates. *igm*: immunoglobulin M, *il-1β*: interleukin 1β, *tnf-α*: tumor necrosis factor α, *jnk1*: c-Jun N-terminal kinase1, *erk1*: extracellular regulated protein kinase 1, *p38α*: mitogen activated protein kinase p38α, *nf-κb1/2*: nuclear factor-κ-gene binding 1/2, *pi3 k*: phosphatidylinositol 3-kinase, *akt*: protein kinase B, *myd88*: myeloid differentiation factor 88, *traf6*: tumor necrosis factor receptor-associated factor 6, *tlr1/2/3/4/5*: toll-like receptor 1/2/3/4/5. The values of *p* < 0.05 and 0.01 are represented as “*” and “**” above the column, indicating significant difference versus control, respectively.

Levels of innate immune parameters (TNF-α, IL-1β) and acquired immune parameter (IgM) in the spleen and head kidney decreased along with the increase in ammonia exposure levels (Figure 6, Supplementary Table S1). In the spleen, the contents and mRNA levels of TNF-α and IgM were significantly decreased in all ammonia treatment groups compared with the controls (*p* < 0.05). Meantime, splenic IL-1β content and mRNA expression levels were significantly down-regulated in the fish exposed to greater than 20 mg/L total ammonia nitrogen (*p* < 0.05). By contrast, only the content and mRNA level of IL-1β as well as the expression level of *tnf-α* in the head-kidney were markedly reduced in ammonia treatment groups relative to controls (*p* < 0.05). There were no significant differences in the levels of IgM protein and gene expression as well as TNF-α concentration in head kidney between ammonia-treated groups and the control group (*p* > 0.05).

To elucidate the molecular mechanism of chronic ammonia toxicity on the immune system, transcriptional changes of key genes involved with the TLR signaling pathway were determined along with the levels of TNF-α, IL-1β and IgM. In the spleen, mRNA levels of toll-like receptor genes (*tlr2*, *tlr4*), MyD88-independent pathway associated gene *traf6*, NF-κB signaling pathway associated gene *nf-κb2* and MAPK signaling pathway associated gene *erk1* were significantly down-regulated in the ammonia treatment groups compared with the control (*p* < 0.05), while there were no remarkable changes in the expression of *tlr1*, *tlr3*, *tlr5*, *pi3 k*, *akt*, *myd88*, *nf-κb1*, *jnk1* and *p38 a* (*p* > 0.05). Similarly, transcription levels of *tlr1*, *tlr2*, *tlr5*, *akt*, *traf6*, *nf-κb1*, *erk1* and *jnk1* were significantly reduced in the head-kidney of fish following chronic ammonia exposure (*p* < 0.05). However, there were no significant differences in expression of *tlr3*, *tlr4*, *myd88*, *pi3 k*, *nf-κb2* and *p38α* in the head-kidney between ammonia treatment groups and the control group (*p* > 0.05).

### 2.6. Correlation Analysis

**Figure 7:**
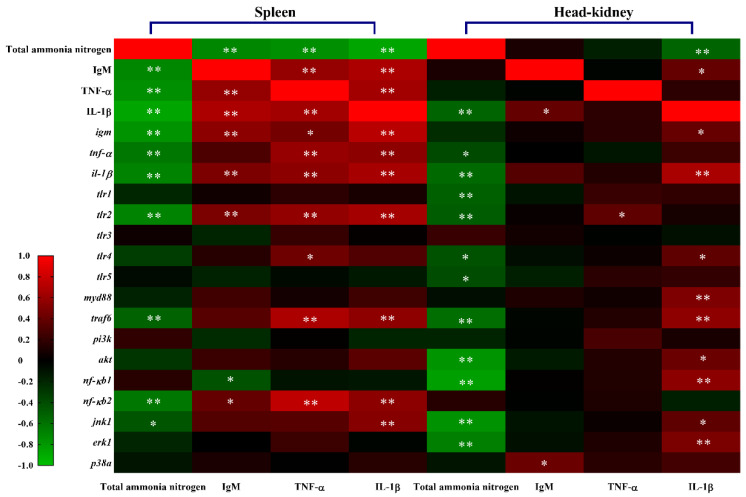
Spearman correlation coefficients ® among ammonia exposure concentration and immune parameters involved with TLR signaling pathway in the spleen and head-kidney of *M. amblycephala* following chronic ammonia exposure. The values of *p* < 0.05 and 0.01 are represented as “*” and “**” above the column, indicating significant difference versus control, respectively.

As shown in Figure 7 and Supplementary Table S2, ammonia exposure concentration was significantly negatively correlated with the contents of TNF-α, IL-1β and IgM as well as transcriptional levels of *tnf-α*, *il-1β*, *igm*, *tlr2*, *traf6*, *nf-кb2* and *jnk1* in the spleen. Meanwhile, the contents and transcription levels of splenic TNF-α, IL-1β and IgM exhibited significant positive correlations with each other (*p* < 0.05). Splenic IgM concentration showed a significant positive correlation with *tlr2* and *nf-кb2*, and TNF-α as well as IL-1β exhibited significant positive correlations with *tlr2*, *traf6* and *nf-кb2* (*p* < 0.05). However, in the head-kidney, ammonia exposure levels were only remarkably negatively correlated with IL-1β content and the expression of genes *tnf-α*, *il-1β*, *tlr1/2/4/5*, *traf6*, *akt*, *nf-кb1*, *jnk1* and *erk1* (*p* < 0.05). Head-kidney IL-1β was positively correlated with IgM, *igm*, *il-1β*, *tlr4*, *myd88*, *traf6*, *akt*, *nf-кb1*, *erk1* and *jnk1*, while IgM and TNF-β exhibited a positive correlation with *p38 a* and *tlr2*, respectively (*p* < 0.05).

### 2.7. IBR Indices

**Figure 8:**
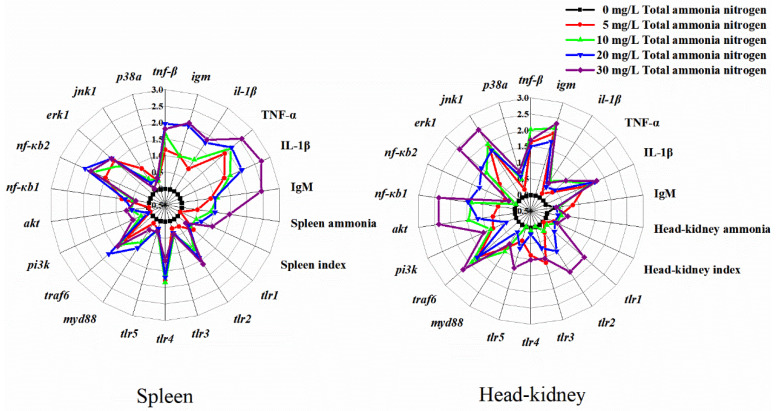
Star plots for biomarker responses in spleen and head-kidney in *M. amblycephala* exposed to ammonia nitrogen.

IBR values were calculated from the standardized data of 22 biomarkers in spleen and head-kidney in Wuchang bream under different levels of ambient ammonia after 30-day exposure (Table 2, Figure 8). As the exposure concentrations of ammonia increased, the IBR values tended to increase in both spleen and head-kidney. The values of IBR were higher in the spleen than in the head-kidney when the fish were exposed to the same treatment conditions (Table 2).

## 3. Discussion

How the immune system of fish responds to different kinds of environmental pollutants has become a hot topic [34,35,36,37]. As a ubiquitous toxicant, ammonia may have an unexpected impact on aquatic animal health since fish often suffer from chronic ammonia stress in realistic situations. Our present study clarified the influences of long-term exposure to ammonia on fish immunity and its mechanism.

In our present experiment, serum cortisol increased significantly with the increase in ammonia exposure concentration, indicating the occurrence of stress in Wuchang bream. Similar results were observed in turbot (*Scophthalmus maximus*), common carp (*Cyprinus carpio*), and juvenile blunt snout bream (*Megalobrama amblycephala*) [32,38,39]. Chronic ammonia exposure also led to significant decreases in serum lysozyme, complement C3 and C4 in this study. Lysozyme, as an indicator of innate immune function, is the primary immune enzyme for fighting infections [40]. The complement system, as one of the bridges between innate immunity and acquired immunity, plays essential roles in clearing immune complexes, killing disease-causing bacteria and viruses [41,42]. Some previous studies have reported that acute ammonia stress can inhibit lysozyme activities [17,43] and decrease protein and mRNA levels of C3 and C4 in fish [44,45,46]. Thus, our present results revealed that chronic ammonia stress subverted the innate immunity of fish.

In the present study, concentration-dependent increases in ammonia concentrations were found both in the spleen and head-kidney, but a significant difference was only detected in the spleen. Similar increases were found in the spleen and head-kidney indexes, indicating that ammonia stress caused severe damage to two main immune organs. Histological findings further revealed that the accumulation of ammonia might be the main cause of pathological injuries in the spleen and head-kidney, which were characterized by increased melano-macrophage centers and cytoplasm vacuolization. Melano-macrophage centers are known as macrophage aggregates, and their main functions are in the storage of cell-derived phospholipid and iron following erythrophagocytosis, deposition of resistant pathogens, and antigen processing in immune responses [47,48,49,50,51]. The increased size and frequency of melano-macrophage centers often indicates that fish are experiencing stress or damage or growing older [36,48,52]. The research of Kwon and Chang reported that a 5-day ammonia exposure (4.0~10.4 mg/L total ammonia nitrogen) induced severe hemosiderin deposition and increased melanin-macrophages in the spleen of black seabream [53]. Similar melano-macrophage assembly was observed in the spleen of *Pelteobagrus vachellii* exposed to 1 and 5 mg/L total ammonia nitrogen [17]. In addition, quantitative evaluation of histopathological alterations showed that the degree of pathological injury caused by persistent ammonia stress was higher in the spleen than in head-kidney.

Immune organ damage in fish is always associated with alterations of immune molecules [35]. In the innate immune system, the inflammatory cytokines IL-1β and TNF-α are the important regulators of the initiation and modulation of inflammatory response [54,55,56,57]. Decreased levels of cytokine molecules (TNF-α, IL-1β) represent a reduction of cellular immunological function, which ultimately results in a high infection rate under external stressors [58,59,60]. Limited studies have documented that acute exposure to 40 mg/L total ammonia nitrogen can elevate transcriptional levels of cytokines such as IL-1β and TNF-α in turbot (*Scophthalmus maximus*), whereas chronic exposure to high ammonia (50 mg/L total ammonia nitrogen) induced the opposite trend in crucian carp (*Carassius auratus*) [14,60]. Our present results showed that chronic ammonia exposure caused marked decreases in protein and transcriptional levels of splenic IL-1β and TNF-α, which were further supported by the Spearman correlation analysis between ammonia concentrations and the levels of splenic IL-1β and TNF-α. Similar results were also found in the head-kidney. Our current data indicated that chronic ammonia exposure disrupted the innate immune defense by inhibiting transcription and protein synthesis of inflammatory molecules IL-1β and TNF-α in two immune organs, the spleen and head-kidney.

In addition to the innate immune defense system, adaptive immunity also plays a crucial role in fish immune defense. IgM is the first antibody secreted by the adaptive immune response to a new infection or to a foreign antigen, which are mainly expressed in the spleen and head-kidney of fish [61,62]. Lower levels of IgM may be associated with weak adaptive immunity [63]. In the present study, significant decreases in protein and transcriptional levels of IgM were detected in the spleen but not in the head-kidney, indicating that ammonia exposure has an inhibitory effect on the production of splenic IgM. Qin et al. reported that exposure to 1 mg/L total ammonia nitrogen for 48 h and 96 h significantly decreased transcriptional levels of *igm* in the spleen of *Pelteobagrus vachellii*, but not in the head-kidney [17]. Decreased IgM levels were also found in the gill, spleen and brain of *Rhynchocypris lagowski* exposed to 0.99 mg/L un-ionized ammonia for 96 h [18]. Our Spearman correlation analysis further showed that ammonia exposure concentration was significantly negative correlated with splenic IgM, but not significantly correlated with head-kidney IgM. Thus, our study suggested that chronic ammonia stress diminished the adaptive immunity only in the spleen. That might suggest that the spleen is more vulnerable to ambient ammonia stress than the head-kidney. One reason for this hypothesis may be the discrepant impairment of the structure of the spleen and head-kidney induced by ammonia stress in our present study.

The TLRs widely distributed in immune cells are primary sensors of invading pathogens. In the present study, decreased transcriptional levels of TLRs were detected in the spleen and head-kidney of Wuchang bream, implying that ammonia could cause negative effects via the TLR signaling pathway. Indeed, the inhibition of TLRs might be related to the increases in serum cortisol induced by ammonia exposure. Carrizo et al. proved that cortisol treatment decreased mRNA levels of TLRs (*tlr1/5 m/9/22*) in *Oncorhynchus mykiss* myotubes [64]. Susarla et al. found that cortisol could decrease transcriptional levels of cytokines (VEGF, CCL5, IFN-γ, CXCL-10, IL-8 and GCSF) after either or both TLR3 and TLR4 stimulation of primary human corneal fibroblasts [65]. Furthermore, the TLR inhibition might imply a block of TLR signaling pathways. The MyD88-dependent pathway is regulated by all TLRs, and the MyD88-independent pathway is peculiar to the TLR3 and TLR4 signaling pathway [66]. TLR signaling also leads to PI3 K-AKT pathway activation, which in turn activates B cells to produce antibody and synthesize proinflammatory molecules such as IL-1β and TNF-α through the NF-κB signaling pathway [67,68,69]. It is worth noting that transcriptional levels of *traf6* were decreased in both spleen and head-kidney after ammonia exposure, while no significant changes were detected in the expression of *myd88* and *pi3 k*. Thus, our results indicated that ammonia exerted interference with the MyD88-independent pathway by inhibiting TRAF6 in both spleen and head-kidney. Additionally, the TRAF6 activation can trigger the activation of downstream NF-κB signaling and MAPK signaling (p38, JNK and ERK), which ultimately induces the production of various inflammatory cytokines including TNF-α and interleukin-1β/6/12 (IL-1β/6/12) [70,71]. In addition, TRAF6 is also the crucial mediator for CD40 signaling that regulates IL-6 and Ig secretion [72]. In our study, the significant down-regulation of splenic *erk1* and *nf-κb2* mRNAs as well as head-kidney *erk1*, *jnk1* and *nf-κb1* mRNAs suggested that ammonia exposure suppressed the downstream NF-κB and MAPK signaling molecules in the spleen and head-kidney. Moreover, ammonia exposure down-regulated NF-κB signaling by suppressing gene *nf-κb1* in the spleen but gene *nf-κb2* in the head-kidney, which reflects the tissue-specific inflammatory response between spleen and head-kidney. As for the MAPK signaling pathway, ammonia exposure significantly inhibited the expression of splenic *erk1* and head-kidney *erk1* and *jnk1*. After performing Spearman correlation analysis, we found that ammonia exposure concentration was significantly negatively correlated with the levels of immune parameters (IL-1β, TNF-α and IgM) as well as the expression of TLR signaling-related genes in the spleen and head-kidney. Therefore, our results suggested that chronic ammonia exposure could impair innate and adaptive immunity via the TLRs/MyD88-independent signaling pathway.

High IBR values reflected the enhanced biological responses and poor health condition of the organisms [73]. The present results for IBR values increased with the increases in ambient ammonia concentration in both spleen and head-kidney. This result was consistent with the data on histopathology and serum-immune parameters. Moreover, under the same concentration of ammonia stress, the IBR value was higher in spleen than in head-kidney, which further proved that the influences of ammonia stress on the spleen were more severe than on the head-kidney of Wuchang bream.

## 4. Materials and Methods

### 4.1. Animal Maintenance and Experimental Protocol

Juvenile Wuchang bream with a mean weight of 9.98 ± 0.48 g from Tuanfeng Fishery (Hubei, China) were moved to 300 L fiberglass tanks, where they were supplied with running de-chlorinated and continuously aerated water. They were acclimated for 14 d by feeding commercial diets twice a day (9:00 am and 15:00 pm). After a preliminary experiment, the 96 h LC50 and safe concentrations of total ammonia nitrogen for bream juveniles were found to be 46.013 mg/L and 4.601 mg/L, respectively. Therefore, the exposure range of ammonia was set as 0 mg/L, 5 mg/L, 10 mg/L, 20 mg/L and 30 mg/L total ammonia nitrogen. Fish were randomly distributed into 5 treatment groups with triplicate tanks, and the stoking density was 20 juveniles per tank. The experimental concentrations of ammonia were obtained from a stock solution of 10 g/L made with reagent-grade ammonia chloride (BASFR, 99.5%). To maintain stable ammonia treatment concentrations, the 1/2 experiment solution was renewed every day by a new and equal concentration of ammonia nitrogen solution. Meanwhile, the control group underwent similar manipulation with aerated tap water. The real total ammonia nitrogen levels for each treatment were measured by nesslerization every day. During the whole experimental period, the daily monitoring data by HQ40 D Water Analyzer (Hach, Loveland, CO, USA) showed that water temperature was 25.0 ± 0.60 °C, dissolved oxygen concentrations were above 5.0 mg/L, and pH was kept at 7.55 ± 0.04, with slight adjustments using 10% H_2_SO_4_ and 10% NaOH. Fish were fed a commercial diet twice per day as same as the acclimation.

### 4.2. Sample Collection and Preparation

After a 30-day experimental exposure, the fish were fasted for 48 h firstly and then were anesthetized in MS-222 solution. The body weight of each juvenile was measured before dissection, and the blood was collected immediately from the caudal veins to separate the serum for subsequent biochemical parameter determination. The tissues (spleen and head-kidney) were extracted and weighed. The spleen and head-kidney indexes were calculated using the formula [weight of tissue (g)/body weight (g) * 100%]. Three individual spleens and head-kidneys from each group were used for histopathological analysis, and the others were frozen immediately at −80 °C for the analysis of tissue ammonia concentrations, immunity parameters and gene transcriptional levels. This work conducted on *M. amblycephala* was approved by the Animal Care and Use Committee (IACUC) of Huazhong Agricultural University, Wuhan, China (HZAUFI-2019-018).

### 4.3. Ammonia Detection in Spleen and Head-Kidney

For the analysis of ammonia levels, the pretreatment of tissue samples and ammonia detection was carried out according to previous studies [74,75]. The detailed test steps can be found in Supporting Information (Supplementary Text S1).

### 4.4. Serum Immune Parameters Assay

Blood samples were centrifuged (845 g, 20 min, 4 °C) for preparation of serum samples. Each experimental group had six replicates and one replicate included the serum from 10 individuals of the same tank. Serum cortisol was determined by radioimmunoassay (RIA) using the kit of Beijing North Institute of Biotechnology Co., Ltd. (Beijing, China) (www.bnibt.com, accessed on 15 March 2020) according to previous study [76]. The contents of complement C3 and complement C4 and the activity of lysozyme were measured using commercial kits produced by Jiancheng Bioengineering Institute (Nanjing, China) (www.njjcbio.com, accessed on 15 March 2020).

### 4.5. Tissue Immune Parameters Assay

Tissue samples from five fish of the same group were pooled as one duplicate and homogenized with 0.85% sodium chloride, then centrifuged at 845× *g* for 15 min (4 °C) for collection of supernatant. Each experimental group had six replicates. As described in previous studies [77,78], supernatant was used to test the concentrations of IgM, IL-1β, TNF-α and total protein using commercial kits. All kits were purchased from Jiancheng Bioengineering Institute (Nanjing, China) (www.njjcbio.com, accessed on the 15 March 2020).

### 4.6. Gene Expression Analysis

The Mrna levels of immune-associated genes in the spleen and head-kidney were tested according to the method in a previous study [79]. Detailed information on the testing procedure is provided in Supporting Information (Supplementary Text S2). The primer sequences are listed in Table 3. Melt curve analysis was performed for each primer at the end of the reaction to demonstrate the reaction specificity. After verifying that the amplification efficiencies of all selected genes ranged from 90–110%, the relative Mrna levels were calculated by the method of 2^−∆∆Ct^ with *β-actin* as the internal control [80].

### 4.7. Histopathological Evaluation

Spleen and head-kidney samples were fixed in 10% neutral-buffered formalin, then dehydrated in ethanol and embedded in paraffin. Finally, tissue slices were stained with hematoxylin and eosin (H&E). Further quantitative analysis of pathological changes was performed according to previous studies [82,83]. The detailed analysis protocol is supplied in Supporting Information (Supplementary Text S3).

### 4.8. Integrated Biomarker Response Analysis

IBR analysis is a method for integrating all of the measured biomarker responses into an integrative index to assess stress levels [84,85,86]. In the current study, it was applied to evaluate the toxic effects of different ammonia concentrations on the spleen and head-kidney of Wuchang bream after a 30-day exposure. Detailed information on the calculation procedure is provided in Supporting Information (Supplementary Text S4).

### 4.9. Statistical Analyses

All values were subjected to one-way analysis of variance followed by Dunnett’s post hoc test to evaluate differences between means (SPSS 22.0, Chicago, IL, USA). Spearman correlation analysis was chosen to determine the relationship among total ammonia concentrations, immune parameters and gene expression levels. Normality and variance homogeneity were previously verified. Differences were measured and considered to be significant at the *p* value < 0.05.

## 5. Conclusions

Our study provides evidence that chronic ammonia stress elevated serum COR, reduced serum lysozyme and C3/C4, and decreased the protein and transcriptional levels of IL-1β, TNF-α and IgM as well as the expression levels of genes involved with TLRs/MyD88-independent signaling pathway in the spleen and head-kidney of *M. amblycephala*. In addition, the indexes of and pathological damage to the two immune organs increased with tissue ammonia accumulation. These findings indicated that ammonia accumulation in the spleen and head-kidney caused histopathological damage and induced immune suppression through inhibition of TLRs/MyD88-independent signaling. In addition, the adverse influences of chronic ammonia stress on the spleen were shown to be more severe than those on the head-kidney of Wuchang bream.

## Figures and Tables

**Table 1:** Quantitative analysis of histopathological changes in the spleen and head-kidney of *M. amblycephala* under chronic ammonia exposure.

		Ammonia Nitrogen Concentration (mg/L)				
Tissue	Lesions	0	5	10	20	30
Spleen	Melano-Macrophage centers	0.00 ^a^ ± 0.00	0.00 ± 0.00	1.00 ± 0.58	1.33 ± 0.67	1.33 ± 0.33
	Increased erythrocytes	0.00 ± 0.00	2.67 ± 0.88 *	5.33 ± 0.67 **	6.00 ± 0.00 **	6.00 ± 0.00 **
	Cytoplasm vacuolation	0.00 ± 0.00	0.00 ± 0.00	0.00 ± 0.00	0.00 ± 0.00	0.67 ± 0.33 *
Head-kidney	Melano-Macrophage centers	0.00 ± 0.00	0.00 ± 0.00	0.67 ± 0.67	0.67 ± 0.33	2.33 ± 0.67 *

^a^: Six different grades (0 unchanged–6 severe) indicated increased levels of histopathological damage. All values are expressed as mean ± SE (*n* = 3). The values of *p* < 0.05 and 0.01 are represented as “*” and “**” above the column, indicating significant difference versus control, respectively.

**Table 2:** Integrated biomarker response (IBR) indices of all measured parameters in the spleen and head-kidney of *M. amblycephala* to ammonia.

	IBR/n	
Ammonia Nitrogen (mg/L)	Spleen	Head-Kidney
0	0.00	0.00
5	0.34	0.34
10	0.48	0.47
20	0.69	0.42
30	0.87	0.95

**Table 3:** Sequences of primers used for Qpcr amplification.

Target Gene	Primer Sequences (from 5′ to 3′)		Accession Number and/or References	Amplification Efficiency
*igm*	F: TGGAGCAACGGCACAGTATT	R: CTCTTGGGACTCGCACCATT	KC894945	99.03%
*il-1β*	F: ACGATAAGACCAGCACGACC	R: CTGTTTCCGTCTCTCAGCGT	KF515511	104.87%
*tnf-α*	F: TCCAAGGCAGCCATCCATTT	R: GCCTGAAGAGAAAGCCTGGT	KF515512	103.97%
*jnk1*	F: AGCACCCCTACATCAACGTG	R: CGTTTTTCGTTCGCTCCTCC	MK315047	109.47%
*erk1*	F: TCCTGCGAGGGCTGAAATAC	R: TCCGGTGTGGTCATGTTCTG	MK315044	101.48%
*p38α*	F: TGGGAGCGGATCTCAACAAC	R: TCAGGCCAGCTGAATGGATG	MK315052	91.86%
*nf-κb1*	F: TGGATGGAGGGGCAGATGTA	R: AAGTGCGCTCAGTTTGCTTG	MK315050	108.68%
*nf-κb2*	F: AACTACCAGTTGAGCGGTGG	R: GGTCACTGCAGGATTTCCCA	MK315051	99.24%
*pi3 k*	F: GGCGTAACATCCAGCTTTGC	R: GCTCCTGGAAGCTGGGTAAC	Liang et al. [81]	93.04%
*akt*	F: GCTGGGTAAAGGCACGTTTG	R: CTCTCGGTGACCGTATGAGC	Liang et al. [81]	101.56%
*myd88*	F: TGGAACAGACTGAATACAAC	R: GACAACAGGGATTAGACG	KP192128	109.31%
*traf6*	F: ATCTGAGCCCGACAGAGAAC	R: CGAGCGAAGACCCATTAGAC	KP192129	109.86%
*tlr1*	F: TCCTGGCTGTTACGATTCTG	R: GAGGTTATTGCGTGGTGCTT	KX196269	109.45%
*tlr2*	F: TTACTCCACCTTGGGACCTG	R: CTAAGCCATTCTTGTGAACCA	KX196270	90.03%
*tlr3*	F: TTGTGGAAGACAGCCAACC	R: CGCAAAGCATCAAGTGGAAT	DQ986365	108.82%
*tlr4*	F: TGGTGTCGCTTTGAGTTTGA	R: AAGGTTCCCTGCTCCACTTC	KR092315	91.99%
*tlr5*	F: GGAGGACCATCTTACCAA	R: TGTTCCCTACAACCAGCA	KX196271	97.26%
*β-actin*	F: ACCCACACCGTGCCCATCTA	R: GGACAATTTCTCTTTCGGCTG	AY170122	108.48%

## Data Availability

All datasets generated for this study are included in the article/Supplementary Materials.

## Supplementary Materials

The following supporting information can be downloaded at: https://www.mdpi.com/article/10.3390/ijms23063129/s1.
