# Supplementary material for: Evaluation of Ammonia Nitrogen Exposure in Immune Defenses Present on Spleen and Head-Kidney of Wuchang Bream (Megalobrama amblycephala)
Source: Int J Mol Sci. 2022 Mar 15;23(6):3129. doi: 10.3390/ijms23063129 (PMC8953400; doi:10.3390/ijms23063129)
Supplement: Supplementary file 1 [file ijms-23-03129-s001.zip › ijms-1633035-supplementary.pdf]

Supporting information:

Evaluation of ammonia exposure in immune defenses present on spleen and head-kidney of Wuchang bream (*Megalobrama amblycephala*)

Honghui Guo[1], Siqi Chen[2], Kang Ouyang[1], Yu Kuang[1], Hui Yang[1], Yingying Wang[1], Rong Tang[1,3,4,5], Xi Zhang[1,3,4,5], Dapeng Li[1,3,4,5], Li Li[1,3,4,5,*]

[1] College of Fisheries, Huazhong Agricultural University, Wuhan 430070, P.R. China

[2] Hubei Aquaculture Technology Extension Center (Hubei Aquatic Breeds Introduction and Breeding Center), Wuhan 430060, P.R. China

[3] Engineering Research Center of Green development for Conventional Aquatic Biological Industry in the Yangtze River Economic Belt, Ministry of Education, Wuhan 430070, P.R. China

[4] Hubei Provincial Engineering Laboratory for Pond Aquaculture, Wuhan 430070, P.R. China

[5] Freshwater Aquaculture Collaborative Innovation Center of Hubei Province, Wuhan 430070, P.R. China

Correspondence: Dr. Li Li

E-mail: foreverlili78@mail.hzau.edu.cn

Office tel: 86-27-87282113

Text S1: Ammonia detection in spleen and head-kidney

The frozen tissue samples were weighted, homogenized (1:10, w/v) in a cold (4 °C) 6% trichloroacetic acid (TAC), and then centrifuged at 10,000 for 20 min at 4 °C to obtain the supernatant. The pH of the supernatant was adjusted with 2 mol/L $KHCO_3$. The absorbance at 340 nm (25°C) was monitored using a UV-3100PC spectrophotometer (Mapada, Shanghai, China). Freshly prepared NH_4Cl solution was used as a standard for comparison. The values were expressed as μmol/g wet tissue.

Text S2: Gene transcription analysis

Total RNA was extracted from samples using the TRIzol reagent (TaKaRa, Dalian, China) according to the manufacturer's instructions. Total RNA concentration and purity were determined by a NanoDrop ND-2000 spectrophotometer (Thermo Scientific, Wilmington, DE, USA). RNA Integrity was checked by gel electrophoresis and UV spectrophotometry. Reverse transcription was conducted with 1 μg of total RNA from each sample using PrimeScript™ RT reagent Kit with gDNA Eraser (Takara, Dalian, China). Quantitative real-time PCR was performed with iQ™ SYBR® Green Supermix (Bio-Rad Laboratories, CA, USA) using an iQ5 Multicolor Real-Time PCR Detection System (Bio-Rad Laboratories, Hercules, CA, USA). The components of a 20-μl reaction mixture contained 10 μl iQ™ SYBR® Green Supermix (Bio-Rad Laboratories), 0.8 μl of each of the specific primers, 6.4 μl of Rnase-free water and 2 μl of cDNA template. The amplification protocol was as follows, 95 °C for 5 min, 40 cycles of 95 °C for 15 s, 58 °C for 15 s, and then at 72 °C for 15 s.

Text S3. Histopathological quantification evaluation

Spleen and head-kidney samples were cut as size as 3-5 mm^3 and fixed in 10% neutral-buffered formalin. After 48-hour fixation, they were processed routinely including embedded in paraffin wax, sectioned (5 μm), and stained with haematoxylin and eosin (H&E). Histopathological assessment was done on a light microscopy (Nikon H600L Microscope and image analysis system, Tokyo, Japan). The splenic histological changes were further quantitatively evaluated according to a protocol proposed by Bernet et al. and Corbett et al. [82,83]. Images of spleen and head kidney section were captured at 400 x magnification. Three images per tissue section were randomly selected for quantification analysis. Histological alterationwas quantified using either the numerical occurrence of a particular alterationper unit area, or the percentage cover of alteration depending on the alteration and organ. A severity score value from 0 to 6 was assigned for the degree and extent of each alteration: 0 for unchanged tissue, (1 or 2) for mildly increased melano-macrophage centres, cytoplasm vacuolization and erythrocytes, (3 or 4) for moderately increased melano-macrophage centres, cytoplasm vacuolization and erythrocytes, (5 or 6) for severely increased melano-macrophage centres, cytoplasm vacuolization and erythrocytes. Severity score values are based on numeric quantification (either % cover or number per unit area).

Text S4. Integrated biomarker response analysis

The procedure of IBR calculation was briefly described here: (1) Data were standardized by the formula $Y = (X - m)/s$, where X is the value of each biomarker response, m is the mean value of the biomarker, and s is the standard deviation of the biomarker. (2) Using standardized data, Z was calculated as $Z = Y$ in the case of activation or $Z = -Y$ in the case of inhibition. Thus, the minimum value (Min) was obtained for each biomarker. (3) The score (S) was computed as $S = Z + |Min|$, where $S \geq 0$ and |Min| is the absolute value of Min. (4) Calculation of star plot areas by multiplying the obtained value of each biomarker (Si) with the value of the next biomarker, arranged as a set, dividing each calculation by 2. (5) Summing up all values, and the corresponding IBR value is obtained as $IBR = \{[(S1 \times S2)/2] + [(S2 \times S3)/2] + \ldots [(Sn\text{-}1 \times Sn)/2]\}$. IBR/n = IBR/ the number of test parameters.

Table S1. The levels of TNF-α, IL-1β and IgM as well as mRNA levels of genes involved with TLRs signaling pathway in *Megalobrama amblycephala* under persistent ammonia exposure [a]

Tissue	Parameters	Total ammonia nitrogen (mg/L)				
		0	5	10	20	30
Spleen	*tlr1*	1.03±0.10	1.10±0.19	0.87±0.13	0.88±0.14	0.92±0.10
	tlr2	1.06±0.09	0.99±0.10	0.78±0.08	0.71±0.09*	0.65±0.04**
	tlr3	1.19±0.17	1.20±0.11	1.06±0.14	1.08±0.06	1.25±0.11
	tlr4	1.05±0.07	0.63±0.06**	0.61±0.09**	0.65±0.10**	0.77±0.05*
	tlr5	1.02±0.10	1.12±0.09	1.14±0.26	1.11±0.15	1.01±0.14
	myd88	1.05±0.16	1.01±0.15	0.80±0.14	0.73±0.06	0.93±0.10
	traf6	1.01±0.07	0.75±0.17	0.69±0.02*	0.59±0.04**	0.67±0.04*
	pi3k	1.01±0.06	1.01±0.06	0.99±0.07	1.02±0.03	1.09±0.07
	akt	1.05±0.14	1.05±0.11	0.91±0.04	0.92±0.08	0.88±0.13
	nf-κb1	1.05±0.09	1.19±0.07	1.13±0.09	1.17±0.05	1.12±0.03
	nf-κb2	1.03±0.05	0.72±0.07**	0.64±0.06**	0.58±0.05**	0.62±0.05**
	erk1	1.02±0.11	0.68±0.06*	0.73±0.02*	0.66±0.09**	0.66±0.07*
	jnk1	1.14±0.06	1.37±0.18	1.03±0.07	1.07±0.09	1.13±0.12
	p38a	1.01±0.07	1.08±0.12	0.94±0.0	1.09±0.15	0.92±0.12
	igm	1.16±0.14	0.74±0.09*	0.74±0.14*	0.35±0.10**	0.32±0.06**
	tnf-α	1.22±0.14	0.72±0.06*	0.52±0.18**	0.39±0.09**	0.45±0.14**
	il-1β	1.05±0.14	0.74±0.15	0.62±0.08	0.38±0.13**	0.34±0.06**
	IgM	194.81±15.30	134.95±10.17	132.28±9.12*	119.89±5.60*	38.46±4.08**
	TNF-α	67.35±2.48	39.74±3.01**	34.06±1.91**	32.15±3.22**	26.04±1.07**
	IL-1β	78.40±2.46	53.86±2.01**	50.66±3.34**	44.38±2.36**	34.19±4.06**
Head-kidney	*tlr1*	1.24±0.10	1.23±0.14	1.19±0.13	1.09±0.10	0.69±0.05**
	tlr2	1.30±0.04	1.20±0.15	1.22±0.07	1.35±0.18	0.67±0.05**
	tlr3	1.02±0.10	0.73±0.09	1.02±0.13	0.85±0.04	1.15±0.04
	tlr4	1.11±0.11	1.36±0.15	1.07±0.14	1.13±0.16	0.75±0.06
	tlr5	1.04±0.07	1.12±0.12	1.04±0.10	1.19±0.13	0.64±0.05*
	myd88	1.11±0.16	0.88±0.07	0.84±0.10	1.04±0.11	0.92±0.10
	traf6	1.01±0.05	0.71±0.06**	0.66±0.04**	0.70±0.06**	0.60±0.01**
	pi3k	1.02±0.10	0.88±0.09	0.87±0.05	0.97±0.04	0.83±0.06
	akt	1.01±0.05	0.87±0.04	0.72±0.07**	0.78±0.03**	0.53±0.04**
	nf-κb1	1.01±0.08	0.90±0.07	0.72±0.06**	0.69±0.02**	0.49±0.01**
	nf-κb2	1.01±0.05	1.07±0.06	1.01±0.07	1.23±0.10	1.05±0.06
	erk1	1.00±0.04	0.86±0.06	0.76±0.05**	0.72±0.06**	0.55±0.02**
	jnk1	1.01±0.07	0.56±0.06**	0.54±0.05**	0.59±0.04**	0.41±0.04**
	p38a	1.07±0.20	1.01±0.06	0.92±0.09	1.08±0.09	0.86±0.08
	igm	1.04±0.07	1.00±0.16	1.12±0.08	0.94±0.12	0.90±0.05
	tnf-α	1.08±0.17	0.59±0.06**	0.47±0.04**	0.63±0.08**	0.57±0.05**
	il-1β	1.16±0.08	0.46±0.09**	0.40±0.07**	0.55±0.06**	0.35±0.09**
	IgM	149.27±15.40	122.78±21.09	153.55±7.41	151.02±6.59	151.33±9.82
	TNF-α	36.25±2.68	33.92±4.31	45.45±5.08	41.17±2.65	28.40±2.07
	IL-1β	121.49±11.43	82.78±8.12*	78.19±11.08*	73.10±10.62**	71.21±5.25**

[a]: Compared to the control, "*" and "**" indicates a significant change at $P < 0.05$ and 0.01, respectively.

Table S2. The spearman correlation coefficients (r) among the ammonia concentration, immune parameters and gene expression in TLR signaling pathway in the spleen and head-kidney [a]

	Spleen				Head-kidney			
	Total ammonia nitrogen	IgM	TNF-α	IL-β	Total ammonia nitrogen	IgM	TNF-α	IL-β
Total ammonia nitrogen	1.00	-0.71**	-0.75**	-0.86**	1.00	0.10	-0.16	-0.53**
IgM	-0.71**	1.00	0.58**	0.67**	0.10	1.00	-0.02	0.39*
TNF-α	-0.75**	0.58**	1.00	0.63**	-0.16	-0.02	1.00	0.17
IL-β	-0.86**	0.67**	0.63**	1.00	-0.53**	0.39*	0.17	1.00
igm	-0.69**	0.49**	0.55**	0.65**	-0.23	0.06	0.16	0.40*
tnf-α	-0.62**	0.30	0.59**	0.55**	-0.39*	0.00	-0.11	0.23
li-β	-.77**	0.06**	0.45*	0.72**	-0.55**	0.33	0.13	0.66**
tlr1	-0.20	0.06	0.18	0.10	-0.50**	-0.10	0.22	0.19
tlr2	-.69**	0.48**	0.57**	0.64**	-.48**	0.03	0.37*	0.09
tlr3	0.06	-0.19	0.21	0.02	0.22	0.07	-0.01	-0.08
tlr4	-0.3	0.15	0.43*	0.31	-0.43*	-0.07	0.05	0.27
tlr5	-0.05	-0.18	-0.05	-0.13	-0.40*	-0.16	0.16	0.20
myd88	-0.18	0.25	0.08	0.24	-0.07	0.12	0.06	0.50**
traf6	-0.52**	0.34	0.67**	0.56**	-0.58**	-0.03	0.14	0.56**
pi3k	0.19	-0.22	0.01	-0.18	-0.19	-0.02	0.28	0.10
akt	-0.28	0.23	0.15	0.36	-0.77**	-0.14	0.14	0.42*
nf-κb1	0.15	-0.43*	-0.10	-0.12	-0.83**	0.01	0.13	0.55**
nf-κb2	-0.63**	0.39*	0.74**	0.55**	0.15	-0.01	0.11	-0.16
jnk	-0.45*	0.32	0.34	0.53**	-0.77**	-0.10	0.04	0.37*
erk1	-0.19	0.00	0.23	-0.02	-0.66**	-0.08	0.12	0.48**
p38a	-0.11	0.10	-0.01	0.15	-0.11	0.42*	0.16	0.26

[a]: Analysis was conducted separately with 30 samples. Compared to the control, "*" and "**" indicates a significant change at $P < 0.05$ and 0.01, respectively.
